# Beyond classic dermoscopic patterns of dermatofibromas: a prospective research study 

**DOI:** 10.1186/s13256-017-1429-6

**Published:** 2017-09-20

**Authors:** Awatef Kelati, Nima Aqil, Hanane Baybay, Salim Gallouj, Fatima Zahra Mernissi

**Affiliations:** Department of Dermatology, University Hospital Hassan II, Fez, Morocco

**Keywords:** Dermatofibroma, Variants, Dermoscopy, New dermoscopic patterns, Rare dermoscopic patterns

## Abstract

**Background:**

The usual stereotypical dermoscopic pattern associated with dermatofibromas is a pigment network and central white patch. However, this pattern may be difficult to diagnose in some variant cases. We aimed to describe dermoscopic patterns of dermatofibroma according to its histopathological subtypes, with special emphasis on new and rare dermoscopic features.

**Methods:**

This prospective study, which was conducted between September 2015 and May 2016 in the Department of Dermatology, University Hospital Hassan II, Fez, Morocco, included 100 cases of dermatofibroma confirmed on clinical and histological grounds. Each lesion was scored for classic, previously reported, or new dermoscopic features.

**Results:**

All our Moroccan patients had a dark skin phototype (Fitzpatrick scale types IV and V). A total of 14 morphological dermoscopic structures were distinguished, and 17 dermoscopic patterns were observed, with the most common pattern being the central white patch and peripheral pigment network (21%). New patterns observed in our study were a white ring around an ulceration (6%), a pigment network with a pigmented ring around follicular openings (2%), and a discreet peripheral network and starlike white patch (3%). A patchy network with white patches was significantly noted in atrophic dermatofibroma (*p* = 0.01); vascularization was described in both aneurysmal and hemosiderotic dermatofibromas (*p* = 0.002); and a white ring around an ulceration was noted in aneurysmal dermatofibroma (*p* < 0.001).

**Conclusions:**

We provide a description of dermoscopic patterns of dermatofibroma according to its histological subtypes in a dark skin phototype, along with a new report of a white ring around an ulceration as a significant pattern in aneurysmal dermatofibroma.

## Background

Dermatofibroma (DF) or benign fibrous histiocytoma represents one of the most common skin tumors [1]; it accounts for approximately 3% of the skin biopsy specimens received at dermatology laboratories [2]. It is a neoplasm of the soft tissue and bone composed of fibroblastic and histiocytic components without any nuclear pleomorphism or histological anaplasia [3]. In addition to common fibrous histiocytoma, other variants have been described to date, including aneurysmal, hemosiderotic, cellular, epithelioid, atypical, lipidized, clear cell, palisading, atrophic, keloidal, granular cell, myxoid, lichenoid, balloon cell, and signet ring cell variants, with the possibility of coexistence of histological features of several variants in the same lesion [4, 5]. In its classical form as a small, raised, cutaneous nodule with a red-brown surface located on the limbs, DF is easy to diagnose clinically, but the diagnosis becomes difficult in variants and atypical cases. For this reason, it is important to improve other noninvasive diagnostic tools, especially dermoscopy.

A pigment network and a central white patch have been described as the typical appearance of common fibrous dermatofibromas (CFDFs) visualized by dermoscopy. When present, the pigment network and central white patch allow the examiner to confirm the diagnosis and set a conservative therapeutic approach [6], but in variants and atypical DFs, these features are not usually present. Our objective was to investigate the dermoscopic patterns of DF in our Moroccan population and to describe significant patterns related to different histopathological variants of DF.

## Methods

We conducted a descriptive and analytical study of digital dermoscopic images of DF prospectively collected in the Department of Dermatology of the University Hospital Hassan II of Fez in Morocco during a period of 10 months from September 2015 to May 2016.

### Subjects

Ninety-five Moroccan patients with 100 DFs were enrolled in the study. DF was confirmed either by clinical findings in CFDF with the classic dermoscopic pattern of pigment network and central white patch or on histological grounds in difficult and atypical cases of DF. The excision was also performed if the patient requested a histological confirmation.

### Data analysis

Clinical data were obtained for each patient, including age and sex as well as the phototype, location, and size of the lesions. Dermoscopic images were documented with a digital camera (DermLite, 3Gen Inc., San Juan Capistrano, CA, USA; or Fotofinder, Bad Birnbach, Germany) with or without polarized light and with or without immersion. To avoid collapse of the vessels in the lesions, no pressure was used.

Clinical and dermoscopic data were analyzed and evaluated by two independent dermoscopists experienced in dermoscopy in our department. Clinical and dermoscopic images of atypical variants of DF confirmed on the basis of histology were retrospectively analyzed after excision. Dermoscopic features were divided into three categories: (1) classic with pigment network and central white patch, (2) previously reported patterns, and (3) new dermoscopic patterns.

Data extraction was performed using Excel software (Microsoft, Redmond, WA, USA). These data were then analyzed using IBM SPSS Statistics version 20 software (IBM, Armonk, NY, USA), and descriptive statistics were expressed as means and percentages. In the univariate analysis, the chi-square test was used to compare these percentages. A *p* value less than 0.05 was considered statistically significant. All subjects were informed of the conditions related to the study and gave their informed consent.

## Results

One hundred lesions in 95 Moroccan patients were evaluated. The average age of the patients was 40.3±8.7 years old (range 17–56 years old), and there was a female predominance in our sample (63%). All patients had dark skin based on Fitzpatrick skin phototypes (94% phototype IV, 6% phototype V).

The lesions were located on the limbs in 75% of cases and on the back in 20% of cases. The lesions ranged in size from 0.5 cm to 10 cm in a case of a giant DF. The color varied from pink to light and dark brown. Twenty-nine percent of the lesions were nodular, and 50% were popular. Sixteen percent of the lesions were atrophic plaques, and 5% were tumors. Four histological subtypes were distinguished: CFDF (72% of cases), atrophic DF (17%), aneurysmal DF (7%), and hemosiderotic DF (4%). Fourteen dermoscopic structures were described in our study: pigment network (79% of cases), white patches (70%), homogeneous pigmentation (36%), white streaks (18%), globule-like structures (18%), peripheral ring (13%), ulceration (6%), brown streaks (6%), ringlike structures and heterogeneous pigmentation (3%), comedo*-*like openings (3%), inverted network (3%), milia-like cysts (2%), and vascular structures (30%) (Table 1).

**Table 1:** Dermoscopic structures of 100 dermatofibromas

Dermoscopic structures	No. (%)
Network	79 (79%)
Peripheral	45.8%
Patchy	17.7%
Total	15.5%
White patches	70 (70%)
Central scarlike	40%
Eccentric multiples	30%
Homogeneous pigmentation	36 (36%)
Central	26.9%
Eccentric	3.3%
Peripheral	5 .8%
Homogenous pink color	21.1%
Globule-like structures	18 (18%)
White streaks	18 (18%)
Peripheral rings	13 (13%)
Around an ulceration	5.5%
Around the follicular openings	2.2%
Simple ring	5.3%
Brown	10%
White	3%
Ulceration	6 (6%)
Brown streaks	6 (6%)
Ringlike structures	3 (3%)
Comedo-like opening	3 (3%)
Negative network-like appearance	3 (3%)
Heterogeneous pigmentation	3 (3%)
Milia-like cysts	2 (2%)
Vascular structures	30 (30%)
Comma-like vessels	23.3%
Dotted vessels	23.3%
Linear	13.3%

**Fig. 1:**
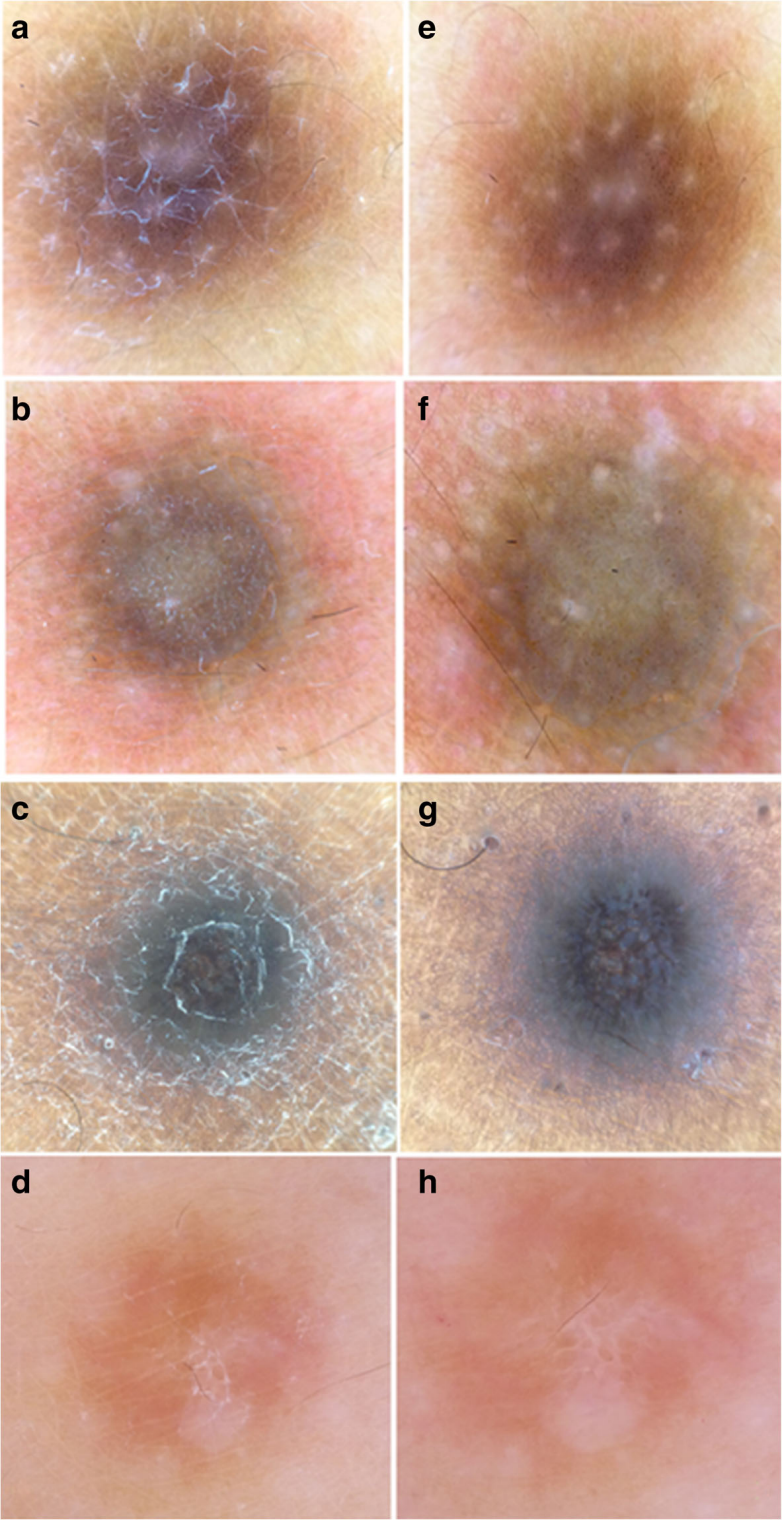
**a**, **b**, **c**, **d** Before immersion. **e**, **f**, **g**, **h** After immersion: better visualization of pigmented structures as peripheral network globules (**e**, **f**), pigmented streaks (**g**), and an inverted network (**h**)

Structures such as an inverted network, a discreet pigment network, globule-like structures, and brown streaks were visualized using immersion (Fig. 1).

**Fig. 2:**
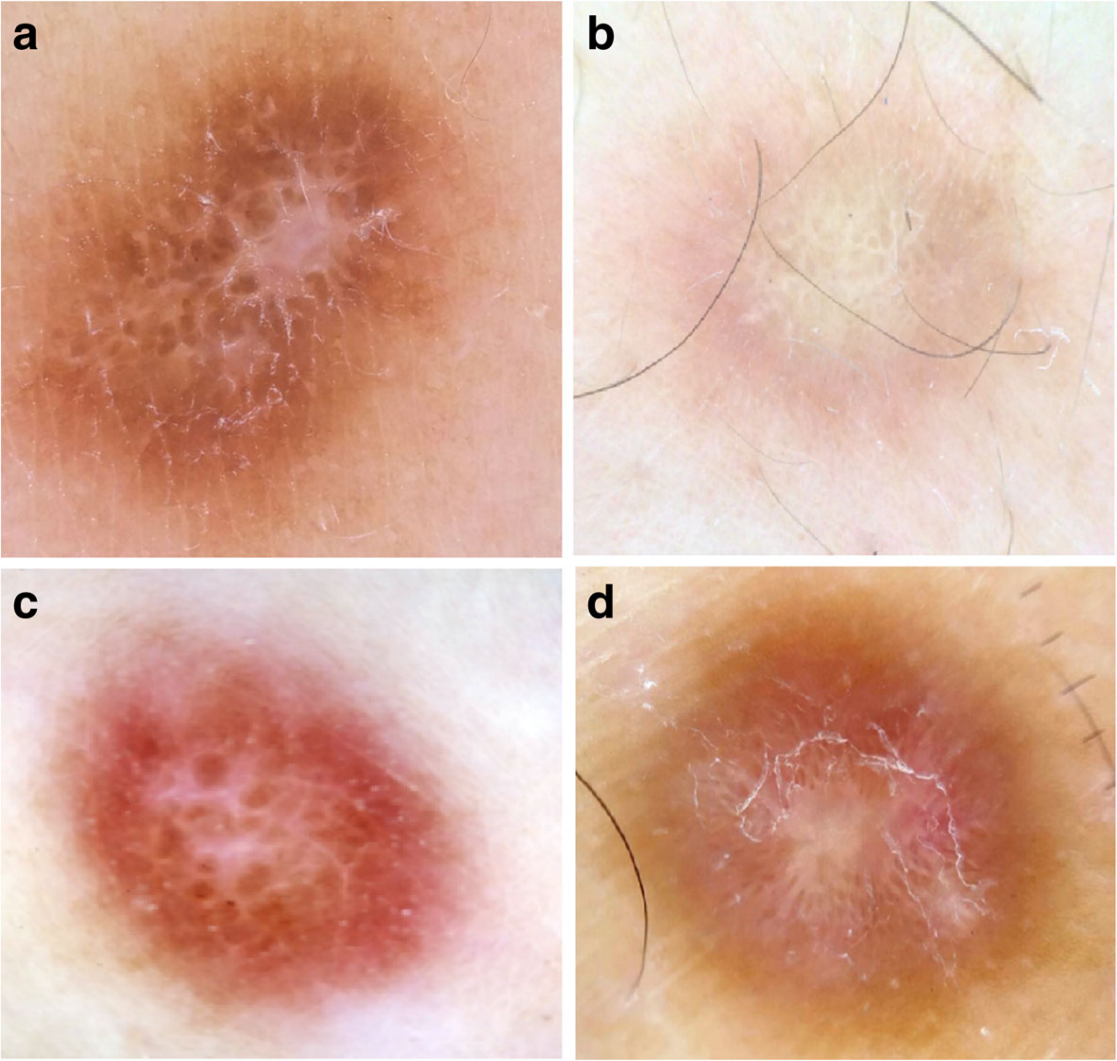
**a**-**d** Classic dermoscopic patterns of common fibrous dermatofibroma: pigment network and central scarlike white patch with central globule-like structures

**Fig. 3:**
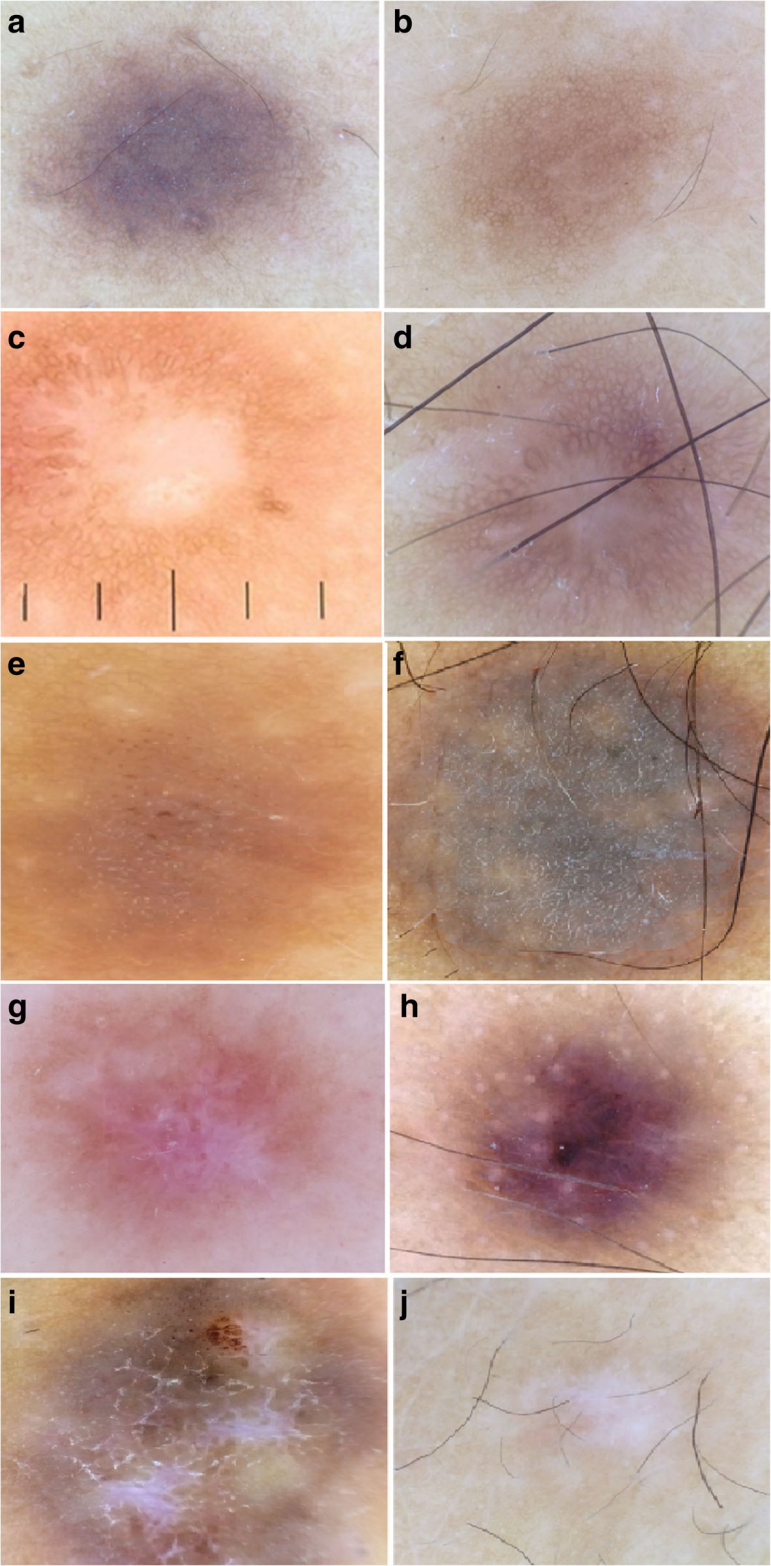
Previously reported dermoscopic patterns of common fibrous dermatofibroma. **a**, **b** Lentigo-like pattern. **a** Dark phototype. **b** Clear phototype. **c**, **d** Peripheral ringlike structures with central white scarlike appearance. **e**, **f** Seborrheic keratosis-like pattern. **g**, **h** Multicomponent pattern. **i** Peripheral pigment network and central inverted network. **j** Total scarlike patch. The vertical lines in panel **c** are the measuring lines of the dermlite dermoscope

**Fig. 4:**
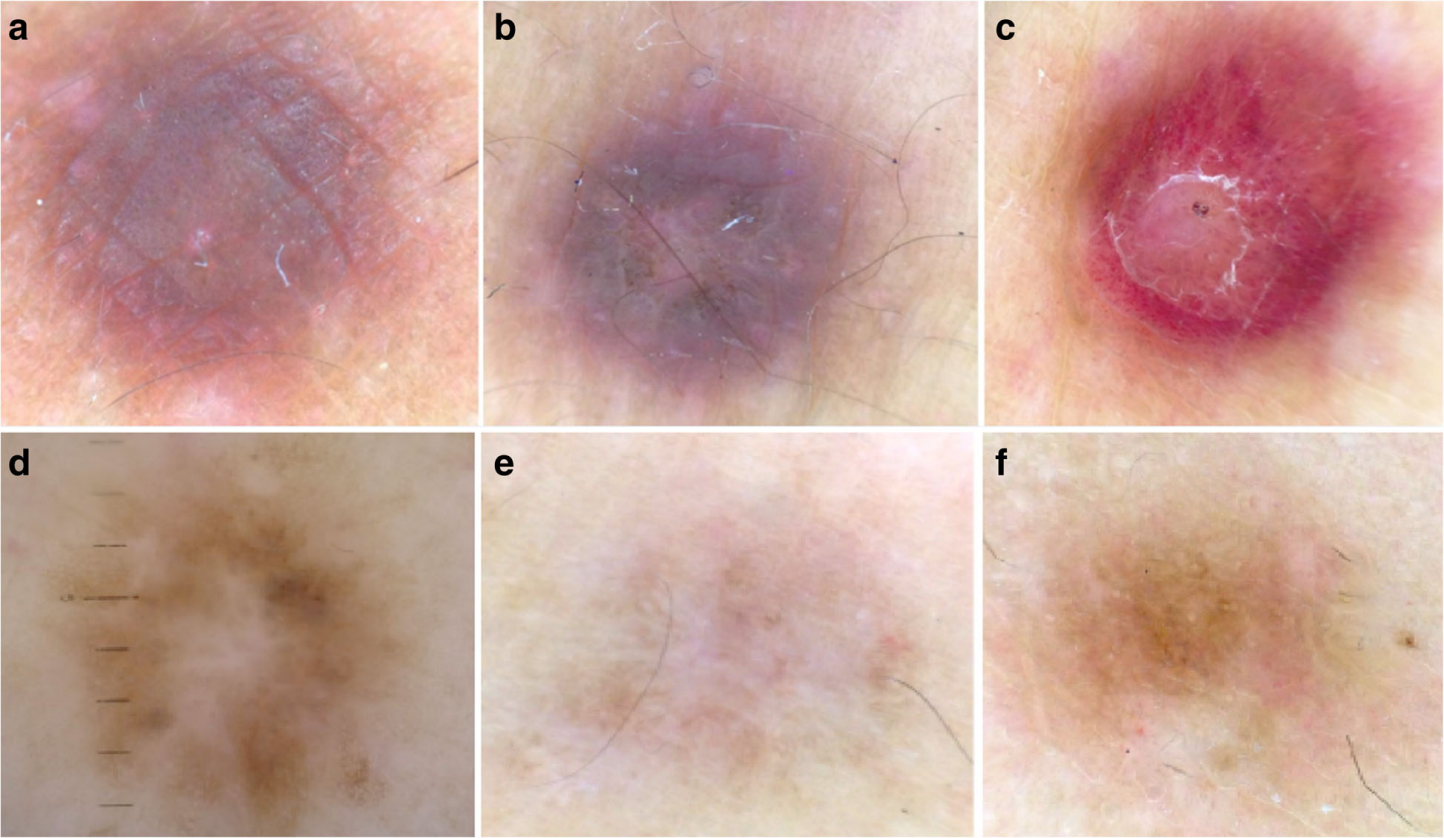
**a**, **b**, **c** Dermoscopic patterns of hemosiderotic dermatofibroma: pink bluish pigmentation with the presence of dotted vessels and comma-like vessels. **d**, **e**, **f** Dermoscopic patterns of atrophic dermatofibroma: patchy network and multiple white patches

**Fig. 5:**
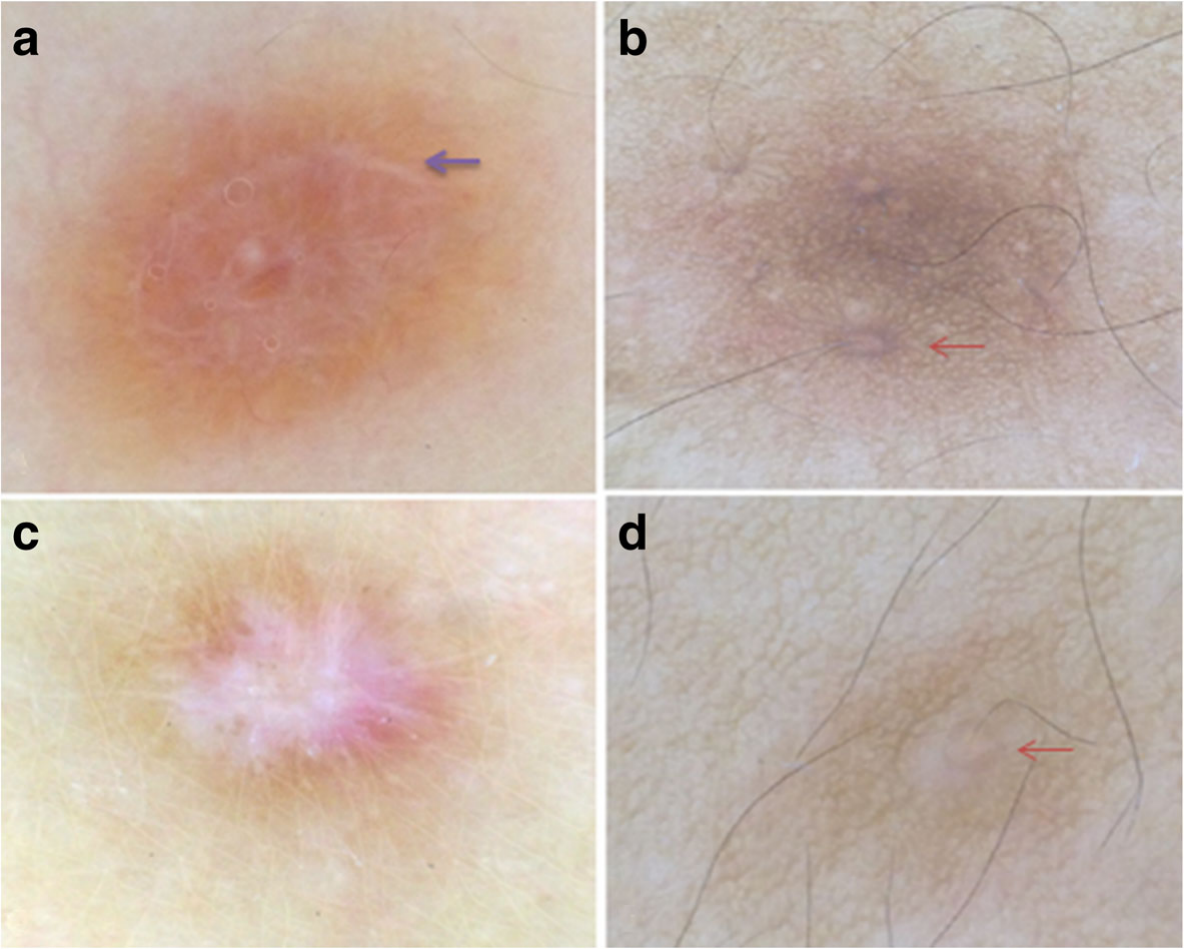
News dermoscopic patterns of CFDF. **a** Total homogeneous pigmentation with white structures forming a ring (black arrow), **b**, **d** Pigmented network with a ring around the follicular openings (red arrow). **c** Discreet peripheral network and star like white patch

**Fig. 6:**
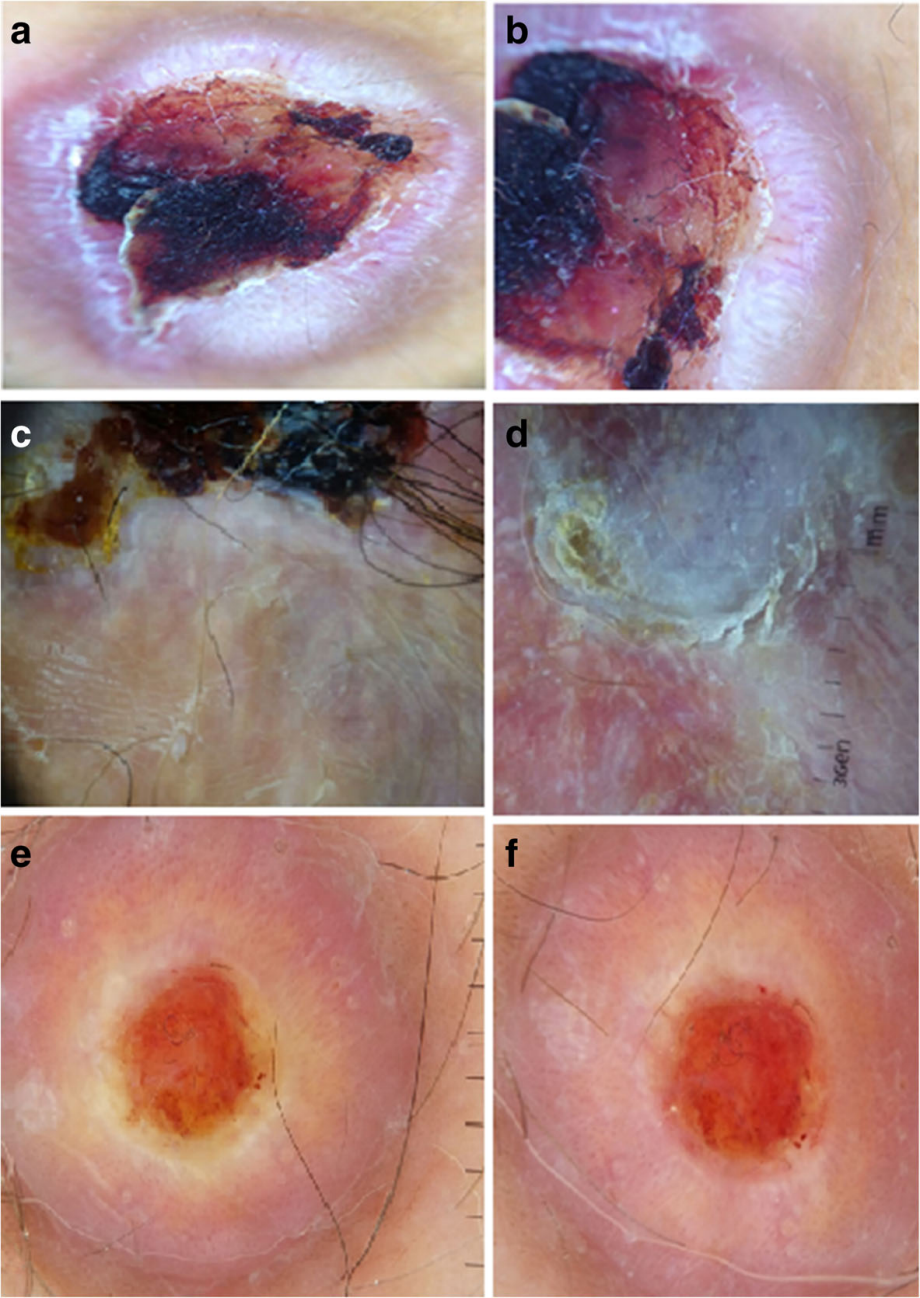
New dermoscopic patterns of aneurysmal dermatofibroma: a-f: white ring around an ulceration with the presence of linear irregular vessels and dotted vessels. The horizontal lines in (**e**) are the measuring lines of the dermlite dermoscope

Seventeen dermoscopic patterns were then distinguished, and they were divided into three categories: classic pattern with pigment network and central white patch (21% of cases), previously reported patterns with multicomponent pattern (20%), patchy network and multiple white patches (16%), lentigo-like pattern (12%), pink bluish pigmentation with vascularization (7%), central homogeneous pigmentation and peripheral pigment network (9%), total homogeneous pigmentation (3%), ringlike structures and a central scarlike white patch (3%), peripheral homogeneous pigmentation and central white scarlike patch (2%), seborrheic keratosis-like pattern (2%), total white scarlike patch (1%), peripheral homogeneous pigmentation and central inverted network (1%), and heterogeneous pigmentation and multiple white patches (1%). New dermoscopic patterns were a white ring around an ulceration (6% of cases), pigment network with a ring around the follicular openings (2%), and a discreet peripheral network and starlike white patch (3%) (Table 2 and Figs. 2, 3, 4, 5 and 6).

**Table 2:** Dermoscopic patterns of 100 dermatofibromas

Dermoscopic patterns	No. (%)
Classic	
Pigment network and central scarlike white patch	21 (21%)
Previously reported	
Multicomponent pattern	20 (20%)
Patchy network and multiple white patches	16 (16%)
Lentigo-like pattern	12 (12%)
Central homogeneous pigmentation and peripheral pigment network with or without white patches	9 (9%)
Pink bluish homogeneous color with vascularization with or without white patches	7 (7%)
Total homogeneous pigmentation with or without white structures	3 (3%)
Peripheral homogeneous pigmentation and central white scarlike patch	2 (2%)
Ringlike structures and central scarlike white patch	3 (3%)
Total white scarlike patch	1 (1%)
Peripheral homogeneous pigmentation or pigment network and central negative network-like	1 (1%)
Seborrheic keratosis-like pattern	2 (2%)
Heterogeneous pigmentation and multiple white patches	1 (1%)
New dermoscopic patterns	
White ring around an ulceration	6 (6%)
Pigment network with a ring around follicular openings	2 (2%)
Discreet peripheral network and starlike white patch	3 (3%)

The aneurysmal variant of DF was significantly noted in men (*p* = 0.03), and it was also related to large lesions (*p* = 0.04). Atrophic DF was significantly localized in the lower limbs (*p* = 0.002). The pink coloration was related to the hemosiderotic DF (*p* = 0.004).

The four patterns significantly related to the classic type of DF were central white patch and peripheral pigment network, homogeneous pigmentation with peripheral pigment network, lentigo-like pattern, and total homogeneous pigmentation (*p* ≤ 0.02), whereas the pattern of patchy pigment network and multiple white patches was significantly described in atrophic DF (*p* = 0.01). Vascularization was described in both the aneurysmal and hemosiderotic DFs (*p* ≤ 0.04), and the aneurysmal DF was characterized by the white ring around an ulceration (*p* < 0.001) (Table 3).

**Table 3:** Univariate analysis showing clinical and dermoscopic characteristics significantly related to different variants of dermatofibroma

Variants of DF	Clinical characteristics	*p* Value	Dermoscopic patterns	*p* Value
CFDF	None		Peripheral pigment network and central white patch	<0.001
			Homogeneous pigmentation and peripheral pigment network	0.005
			Lentigo-like pattern	0.03
			Total homogeneous pigmentation	0.02
Atrophic DF	Localization on the lower limbs	0.002	Patchy pigment network and multiple white patches pattern	0.01
Aneurysmal DF	Male sex	0.03	Vascular structures (linear irregular vessels)	0.04
	Big size of lesions	0.04	White ring around an ulceration	<0.001
Hemosiderotic DF	Pink coloration	0.004	Vascular structures (dots and comma-like vessels)	0.002

*CFDF* Common fibrous dermatofibroma, *DF* Dermatofibroma

## Discussion

DFs may display different morphological faces with different histological variants. In our study, these variants were atrophic DF, aneurysmal DF, and hemosiderotic DF. Although this benign tumor may mimic melanocytic lesions, especially melanoma in its atypical forms [7], the establishment of its significant dermoscopic patterns has developed at a far slower pace than melanocytic lesions because there are fewer than ten analytical studies on DF reported in the literature [8–14], compared with the large number of studies on melanocytic lesions.

The misdiagnosis of DFs as melanocytic lesions is probably more important in our population, owing to the fact that the patterns significantly related to this benign tumor were characterized by pigmented features such as a pigment network, homogeneous pigmentation with peripheral pigment network, a lentigo-like pattern, or total homogeneous pigmentation. This may be explained by the dark skin phototype of our patients, which increased the need for better characterization of DF in the dark phototype.

The pattern of a pigmented peripheral network and a central white scarlike patch was the most stereotypical and common finding of CFDF. It corresponds to pronounced fibrosis within the papillary dermis with hyperpigmented rete ridges at the edge of DF [9]. This pattern represented 62% of cases in the study of Agero *et al.* [14] and 34% in the study of Zaballos *et al.* [15], whereas it was noted in only 21% of cases in our study. This may be explained by the particularity of the skin of our population, as stated above. Wide variation of patterns has been described in the literature and in our work on CFDF, although some of these patterns are very rare, such as the total white scarlike patch and the seborrheic keratosis-like pattern [9, 15, 16]. These must be known as possible presentations of this tumor in order to increase diagnostic accuracy.

Pink bluish pigmentation with vascularization was noted in hemosiderotic DFs in our study and in a case report by Zaballos *et al.* [17], whereas Blum *et al.* [9, 18] reported a pattern of an asymmetric blue grayish area with globules, a pigment network, and streaks without pink bluish coloration. The blue color was reported to correspond to the dermal pigmented siderophages, and the pink color in our study may correspond to the numerous small vessels, extravasated erythrocytes, and hemosiderin deposits [17]. The presence of vascular structures was also noted in aneurysmal DFs, which was similarly reported in one other study [15]. The patchy network and multiple white patches were noted in the atrophic variant of DF. This variant was rarely noted in other studies [14, 15], whereas it represented 16% of cases in our study (Table 4).

**Table 4:** Review of the literature concerning dermoscopic patterns of dermatofibromas

	Zaballos *et al.* [15]: 292 patients 412 lesions	Ferrari *et al.* [10]: 115 patients	Agero *et al.* [14]: 50 patients	Kilinc *et al.* [9]: 52 patients	Present study: 95 patients 100 lesions
Typical					
Peripheral delicate pigment network and central white scarlike patch	143 (34.7%)	23 (17.7%)	31 (62%)		21 (21%)
Total delicate pigment network	60 (14.6%)	4 (3.1%)			
Peripheral delicate pigment network and central white network	37 (9.0%)	6 (4.6%)			
Peripheral delicate pigment network and central homogeneous pigmentation	20 (4.8%)	5 (3.8%)			9 (9%)
Total white network	9 (2.2%)	3 (2.3%)			
Total homogeneous pigmentation	20 (4.8%)	15 (11.5%)			3 (3%)
Total white scarlike patch	23 (5.6%)	0			1 (1%)
Multiple white scarlike patches	24 (5.8%)	5 (3.8%)			16 (16%)
Peripheral homogeneous pigmentation and central white scarlike patch	30 (7.3%)	10 (7.7%)			2 (2%)
Peripheral homogeneous pigmentation and central white network	21 (5.1%)	11 (8.5%)			1 (1%)
Atypical					
Multicomponent melanoma-like	25 (6.1%)	38 (29.2%)			20 (20%)
Vascular tumor-like		21 (16.2%)			
BCC-like		6 (4.6%)			
Collision tumor-like		5 (3.8%)			
Psoriasis-like		3 (2.3%)			
Globules in scarlike area		3 (2.3%)		20 (38.5%)	
Linear, irregular crypts				14 (26.9%)	
Lentigo-like pigment network				12 (23%)	12 (12%)
Homogeneous blue-gray pigmentation				3 (5.9%)	
Erythematous homogeneous area surrounding white patch				2 (3.8%)	
Multiple scarlike structures with pigment network in a patchy distribution (atrophic)				1 (1.9%)	
Central white patch			42 (84%)		
Peripheral pigment network			36 (72%)		
Globule-like structures			22 (44%)		
Blood vessels			22 (44%)		
Central pink hue or vascular blush			5 (10%)		
Peripheral diffuse pink to red to reddish violet halo			14 (28%)		7 (7%)
Peripheral halo of brown pigmentation			2 (4%)		
	Zaballos *et al.* [17] (6 cases)				
Blue violaceous or red bluish homogeneous area with a white linear structure in the center and a delicate light brown pigment network with a few dotted vessels at the periphery	4 Hemosiderotic dermatofibroma				
Blue yellowish homogeneous pattern with scaly surface in the center of the lesion surrounded by a yellowish homogeneous area at the periphery	1 Hemosiderotic dermatofibroma				
Red brownish homogeneous area with a delicate light brown pigment network at the periphery with branched streaks and linear irregular dilated vessels in other central areas	1 Aneurysmal dermatofibroma				
White ring around an ulceration					6 (6%)
Pink bluish pigmentation with vascularization					Aneurysmal dermatofibroma 7 (7%)
Pigment network with a ring around follicular openings					Hemosiderotic dermatofibroma 2 (2%)
Discreet peripheral network and starlike white patch					3 (3%)

*BCC* Basal cell carcinoma

Furthermore, we defined new dermoscopic patterns such as a pigmented ring around the follicular openings in dark-skinned patients. This pigmented ring is different from the small ringlike structures or globule with a darker peripheral rim forming a kind of network, which corresponds to flattened and broad rete ridges, as previously reported by Zaballos *et al.* [15] and Agero *et al.* [14].

The white ring around an ulceration was highly related to aneurysmal DF in this study. It may be a very useful sign to differentiate between aneurysmal DF and achromic melanoma, especially because this variant of DF is also characterized by polymorphous vessels and linear irregular vessels, which may be confusing and misdiagnosed as a malignant tumor. So, in addition to the reported nonspecific signs of a central bluish or reddish homogeneous area with white structures and a peripheral delicate pigment network with vascular structures of variable degrees [9, 19], this ring pattern could be of great help in diagnosing difficult cases of aneurysmal DF.

The white color may be explained by the spindle cells’ disposition and dermal fibrosis around the large blood-filled spaces without endothelial lining [20]. If they are large and superficial, these hemorrhagic spaces may cause epidermal removal and ulceration surrounded by fibrosis, which appears as a white ring around the ulceration.

## Conclusions

This study provides a description of dermoscopic patterns of DF according to histological subtypes in a dark skin phototype, with a new report of patterns such as the white ring around an ulceration as a significant one in aneurysmal DF.
